# Natural Compounds for Wood Protection against Fungi—A Review

**DOI:** 10.3390/molecules25153538

**Published:** 2020-08-02

**Authors:** Magdalena Broda

**Affiliations:** 1 Department of Wood Science and Thermal Techniques, Faculty of Wood Technology, Poznań University of Life Sciences, Wojska Polskiego 38/42, 60-637 Poznań, Poland; magdalena.broda@up.poznan.pl; 2 BioComposites Centre, Bangor University, Gwynedd LL57 2UW, UK

**Keywords:** natural wood preservatives, antifungal properties, essential oils, tannins, propolis, plant oil, plant extracts

## Abstract

Wood is a renewable, versatile material with multiple applications and the largest terrestrial pool of sequestered carbon. However, it is susceptible to degradation, mainly caused by wood-decaying fungi. Since several traditional wood preservatives have been banned owing to their detrimental effects on humans and the environment, extending the lifespan of wood products using new generation natural preservatives is an imperative from the perspectives of human health and environmental protection. Several natural compounds of plant and animal origin have been tested for their fungicidal properties, including essential oils, tannins, wood extractives, alkaloids, propolis or chitosan; and their enormous potential in wood protection has been shown. Although they are not free of limitations, the potential methods to overcome their drawbacks and enhance their bioactivity already exist, such as co-impregnation with different polymers, cross-linkers, metal chelators or antioxidants. The presence of the discrepancies between laboratory tests and the field performance, as well as legislation-related problems resulting from the lack of standards defining the quality and performance of natural protective formulations, however, create an urgent need for further thorough research and arrangements. The collaboration with other industries interested in the utilisation of natural active compounds will reduce the associated costs, thus, will facilitate the successful implementation of alternative antifungal agents.

## 1. Introduction

Wood is a natural, renewable and highly versatile material of excellent performance that has been commonly used by man since the dawn of history. It is also the largest reservoir of sequestered carbon in terrestrial environments. However, its chemical composition and structure make it prone to biodeterioration, and fungi are the main wood degraders [1,2].

Traditionally, regarding the pattern of degradation, three groups of wood-decaying fungi are distinguished, i.e., brown-rot, white-rot and soft-rot (Table 1). All of them degrade structural polymers of the wooden cell wall, which results in the loss of wood strength. Wood can also be attacked by moulds and blue stain (Table 1). Although they do not cause significant structural damage, they adversely affect the aesthetic value of wood since their activity leads to wood discolouration [1,2].

Wood becomes susceptible to fungal infestation under specific environmental conditions, i.e., moisture content above 20%, oxygen availability and a temperature between 15 and 45 °C. Fungal deterioration affects then mainly outdoor wooden structures, reducing wood mechanical and aesthetical properties and significantly limits its service life [5,6]. A broad range of effective synthetic wood preservatives has been applied to prevent this, including copper-based agents (i.e., chromated copper arsenate), triazoles (azaconazole, propiconazole, tebuconazole), pentachlorophenol or boron-based fungicides [7,8,9]. Due to environmental and health concerns, however, many of them have been banned from the use, creating the need for developing alternative wood protection agents and methods based on non-toxic natural products [9,10,11].

Nowadays, environmentally-friendly wood protection is an object of extensive research that covers several different approaches. Since the growth of wood-degrading fungi depends on water availability, one of the methods is moisture control using natural hydrophobising agents, such as resins and waxes of plant or animal origin, or plant oils [12,13,14,15]. Another approach for extending the service life of wood is the utilisation of natural compounds with biocidal properties and fixing them inside the wood structure [11,12,16]. The more innovative method involves using biological control agents, i.e., microorganisms such as other fungi and bacteria which act as antagonists to wood-decaying fungi [12,17].

The review aims to present information about the current research on natural compounds with a proven biocidal activity that can be potentially useful for wood protection against fungi. It is divided into two main parts depending on the origin of the compounds described (plant or animal), and then into subsections regarding the specific source or the type of a substance. The review includes both the results of in vitro studies on antifungal activity of particular natural extracts or their isolated components against wood-inhabiting fungi and the data obtained from mycological tests with the use of wood of different species treated with natural protective formulations. The effectiveness, advantages and disadvantages, as well as problems associated with the use of natural products in wood protection are discussed, showing the potential prospects of their commercial application.

## 2. Antifungal Substances of Plant Origin

Plants are a rich source of various chemical compounds, including alkaloids, flavones and flavonoids, phenolics, terpenes, tannins or quinones. Produced as secondary metabolites, they can constitute up to 30% of the dry mass of plants, playing an essential role in their protection against microbial pathogens, herbivores and different kinds of abiotic stress. Due to their specific properties resulting from the presence of particular phytochemicals, many plants have been used by humans ever since as medicines or food additives. Nowadays, recognising of the chemical structure and functions of particular plant components enables to develop efficient methods of their extraction from plant tissues and use them commercially, i.e., as ingredients of pharmaceuticals, cosmetics, functional food, or colouring agents. There is also a great interest to apply them as biopesticides, insecticides and fungicides to protect crop plants and biodegradable materials [18,19,20,21].

Antifungal properties of various plant extracts make them interesting also as a potential source of natural substances that may be used as alternative wood preservatives against decay. High availability of plant material in general and a prospective possibility of using industrial waste from the processing of different crops can increase the economic viability of the entire process of their obtaining, thus enable for potential widespread application of plant preservatives in the wood industry.

### 2.1. Essential Oils

Essential oils are natural mixtures of volatile secondary metabolites of different plants that can be obtained from raw plant material by distillation, mechanical expression or extraction with the use of different solvents. They contain a variety of chemical compounds that are responsible for a characteristic fragrance of particular plants from which they are derived. The main ingredients are terpenes, including alcohols, aldehydes, hydrocarbons, ethers and ketones, with proved biological activity, such as antioxidant, antibacterial and antifungal. Therefore, plants containing essential oils have been used for centuries in folk medicine and added to food as both flavouring and preservative agents [22,23,24].

Nowadays, essential oils have found application in perfumery, aromatherapy, production of food and cosmetics. Their composition has been extensively studied together with their potential therapeutic activities, including anti-inflammatory, antimicrobial, antiviral, anti-cancer, antidiabetic or antioxidant [23,24,25]. The observed growing interest in bio-friendly, non-toxic natural substances with antimicrobial properties makes essential oils potentially useful as preservatives for a broad range of products [26,27,28]. Due to the proven antifungal properties against mould and wood-decaying fungi, some attempts have also been made to apply essential oils from common plants, herbs and spices as wood protective agents [29,30,31,32,33,34,35].

#### Essential oils in Wood Protection

Several in vitro tests against different fungi species were performed using various essential oils to find the most effective ones. Voda et al. [29] reported high antifungal effectiveness of anise, basil, cumin, oregano and thyme oils against brown-rot fungus *Coniophora puteana* and white-rot fungus *Trametes versicolor* using the agar dilution method. They showed that the most effective compounds in inhibiting the growth of both fungi were thymol, carvacrol, trans-anethole, methyl chavicol, and cuminaldehyde. Their further research confirmed the existence of a relationship between the molecular structure of the oxygenated aromatic essential oil compounds and their antifungal activity against wood-decaying fungi [36]. In vitro tests by Chittenden and Singh [37] demonstrated antifungal effectiveness of 0.5% concentrations of cinnamon and geranium oils against brown-rot fungi *Oligoporus placenta*, *C. puteana* and *Antrodia xantha*, sapstain fungi *Ophiostoma floccosum* Mathiesen, *Ophiostoma piceae*, *Sphaeropsis sapinea* and *Leptographium procerum*, and a mould fungus *Trichoderma harzianum*. They also showed antifungal properties of aniseed, oregano and lema (a blend of 50% New Zealand manuka and 50% Australian tea tree) oils against some of the fungi mentioned above. Zhang et al. [35] reported antifungal effectiveness of pure monoterpenes such as β-citronellol, carvacrol, citral, eugenol, geraniol, and thymol against wood white-rot fungi *Trametes hirsuta*, *Schizophyllum commune* and *Pycnoporus sanguineus*. Xie et al. [34] confirmed antifungal properties of *Origanum vulgare*, *Cymbopogon citratus*, *Thymus vulgaris*, *Pelargonium graveolens*, *Cinnamomum zeylanicum* and *Eugenia caryophyllata* essential oils against wood-decaying fungi *T. hirsuta* and *Laetiporus sulphurous* pointing out carvacrol, citron, citronellol, cinnamaldehyde, eugenol and thymol as the most active compound. Some of the common compounds of natural essential oils, that is cinnamaldehyde, α-methyl cinnamaldehyde, (E)-2-methylcinnamic acid, eugenol and isoeugenol, were shown to effectively inhibit the growth of white-rot fungus *Lenzites betulina* and brown-rot fungus *L. sulphurous* [38]. In turn, the results obtained by Reinprecht et al. [39] show that among five different essential oils (basil, cinnamon, clove, oregano and thyme), the highest antifungal activity against brown-rot fungus *Serpula lacrymans* and the white-rot fungus *T. versicolor* was shown for basil oil (containing manly linalool), and the lowest was noted for clove oil (containing mainly eugenol).

**Figure 1:**
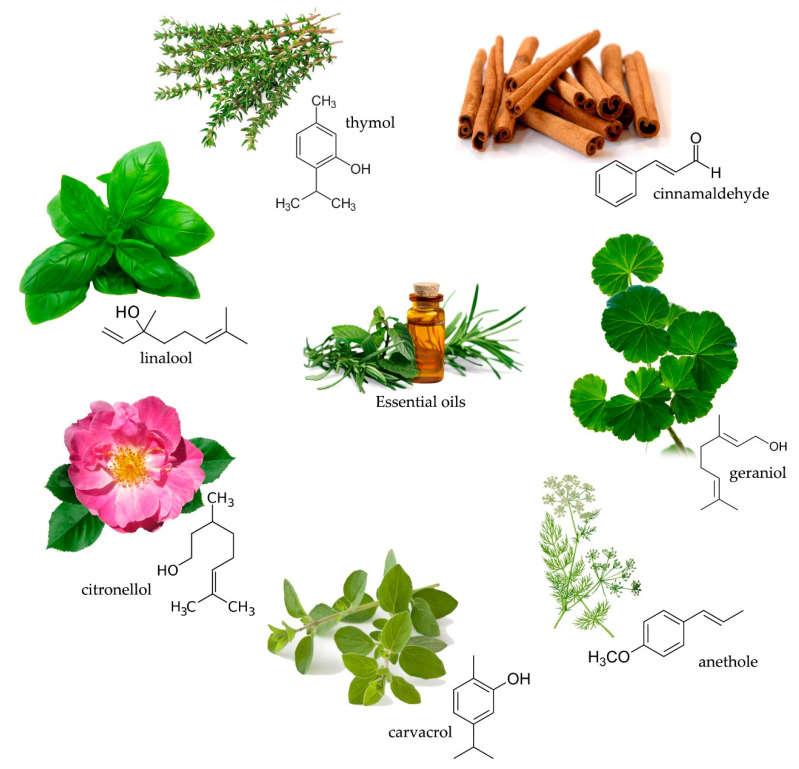
Chemical structure and exemplary plant sources of the selected antifungal compounds of essential oils.

The results mentioned above were confirmed on wood samples treated with selected essential oils. Pánek et al. [33] examined the antifungal effectiveness and stability of beech wood treated with 10% solutions of ten different essential oils (birch, clove, lavender, oregano, sweet flag, savoury, sage, tea tree, thyme and a mixture of eucalypt, lavender, lemon, sage and thyme oils) against brown-rot fungus *C. puteana* and white-rot fungus *T. versicolor*. They found out that after a complex accelerated ageing procedure the most effective against *C. puteana* were clove, oregano, sweet flag and thyme oils that contain phenol compounds such as carvacol, eugenol, thymol and cis-isoasarol trimethylether (chemical structure of the selected compounds of essential oils are presented in Figure 1). Mass losses of birch wood were 0.9%, 0.66%, 0.57% and 0.87%, respectively. Clove, sweet flag and thyme oils were also the most effective against mould (*Aspergillus niger* and *Penicillium brevicompactum*) while tested in filter papers. These oils can be then potentially useful for wood protection in interiors. Interestingly, none of the tested oils was effective against *T. versicolor*, which may result from the specific enzymatic apparatus of white-rot fungi able to degrade both lignin and other phenolic compounds. Effectiveness of thyme oil against *C. puteana* and *A. niger* was also confirmed by Jones et al. [40]. Moreover, they showed antifungal activity of basil, yarrow and calendula oils against *C. puteana* and *P. placenta*, respectively; however, the two latter oils were only effective when used as neat oils. High resistance of radiata pine wood treated with 3% eugenol was reported by Chittenden and Singh [37], with mass losses < 1% when exposed to *C. puteana*, *O. placenta* and *A. xantha*. However, they found out that eugenol can be easily leached out from wood that suggests its unsuitability for protection of wood used outdoors. Kartal et al. [32] treated sugi wood with a formulation containing cassia oil, obtaining high wood resistance against brown-rot *Tyromyces palustris* (mass loss of 0.7%) and white-rot *C. versicolor* fungi (mass loss of 3.6%).

Yang and Clausen studied mould inhibiting properties of seven essential oils including ajowan, dill weed, geranium (Egyptian), lemongrass, rosemary, tea tree and thyme oil. They found out that vapours from dill weed oil and dip treatment of Southern yellow pine samples with thyme or geranium effectively protected wood against the growth of *A. niger*, *Trichoderma viride*, and *Penicillium chysogenum* for at least 20 weeks [41]. The results by Bahmani et al. [31] confirmed that lavender, lemongrass and thyme oils applied for impregnation of *Fagus orientalis* and *Pinus tadea* wood could ensure efficient protection against *A. niger*, *Penicillium commune*, *C. puteana*, *T. versicolor* and *Chaetomium globosum*. The anti-mould activity of *Pinus rigida* and *Eucalyptus camaldulensis* oils applied on *Fagus sylvatica*, *P. rigida* and *P. sylvestris* wood surface was shown by Salem et al. [42] and similar properties of clove oil applied on local Indian timber was reported by Hussain et al. [30].

Great variety of essential oils derived from specific indigenous plants from all around the world were also proved to have protective properties against mould and wood decay. For example an essential oil from the leaves of the Taiwan cinnamon tree *Cinnamomum osmophloeum* Kaneh., containing cinnamaldehyde as the most abundant antifungal component, was reported to be effective against a variety of white- and brown-rot fungi, including *Coriolus versicolor* and *Laetiporus sulphureus* [43]. Antifungal properties of cinnamaldehyde were also confirmed by Kartal et al. [32] when applied for sugi wood treatment, effectively increasing wood resistance against brown-rot *T. palustris* (mass loss of 0.6%) and white-rot *C. versicolor* fungi (mass loss of 3.8%). Good results were also obtained by Chittenden and Singh [37] for radiata pine wood treated with 3% cinnamaldehyde solution, where the mass loss was <1% against *C. puteana* and *A. xantha*, and about 3% against *O. placenta*.

Leaf and fruit oils of another Taiwan tree, *Juniperus formosana* Hayata, was tested in vitro by Su et al. [44] for their antifungal properties against seven mould fungi (*Aspergillus clavatus*, *A. niger*, *Ch. globosum*, *Cladosporium cladosporioides*, *Myrothecium verrucaria*, *Penicillium citrinum*, *T. viride*), two white-rot (*T. versicolor*, *Phanerochaete chrysosporium*) and two brown-rot fungi (*Phaeolus schweinitzii*, *Lenzites sulphureum*). They reported excellent antifungal effectiveness of leaf oil, with α-cadinol and elemol as the most active compounds. High antifungal activity against mould and wood-decaying fungi was also shown for Taiwanese *Eucalyptus citriodora* leaf oil due to the presence of citronellal and citronellol as the main active components [45].

Cheng et al. [46] reported high antifungal activity of essential oil obtained from *Calocedrus formosana* Florin leaves. *C. formosana* is an endemic tree species from Taiwan characterised by natural decay resistance. The strongest antifungal activity against *L. betulina*, *Pycnoporus coccineus*, *T. versicolor*, and *L. sulphurous* were shown for two oil compounds: α-cadinol and T-muurolol.

Mohareb et al. [47] studied the antifungal activity of essential oils from eighteen different Egyptian plants against wood-decaying fungi *Hexagonia apiaria* and *Ganoderma lucidum*. The best resistance was obtained for Scots pine sapwood treated with *Artemisia monosperma*, *Citrus limon*, *Cupressus sempervirens*, *Pelargonium graveolens*, *Schinus molle* and *Thuja occidentalis* oils. In turn, the effectiveness of neem oil, containing azadirachtin as a main antifungal compound, against *S. commune*, *Fusarium oxysporum*, *Fusarium proliferatum*, *C. puteana* and *Alternaria alternate* fungi was reported by Rawat et al. [48]. Similar results were obtained by Hussain et al. [30] who showed resistance of local Indian wood treated with neem oil against various moulds.

Some novel approaches aiming to enhance the effectiveness of antifungal activity of essential oils as wood preservatives are worth to be mentioned here. One of them is using complexes of essential oils with methyl-β-cyclodextrin. Cai et al. [49] treated Southern pine wood with complexes of eugenol, trans-cinnamaldehyde, thymol and carvacrol with methyl-β-cyclodextrin and exposed it to brown-rot fungi *Gloeophyllum trabeum* and *P. placenta*. The results showed an improved decay resistance of wood treated with particular complexes, even after leaching, in comparison with control samples or wood specimens impregnated with essential oils individually. It seems then that the use of specific complexes containing natural compounds such as essential oils has a great potential in extending the lifespan of wood products.

### 2.2. Tannins

Tannins are natural compounds produced by most higher plants to protect them against pathogenic bacteria, fungi and insects. They can be found in almost all parts of a plant, starting from roots, through wood and bark to leaves and seeds [50,51].

Differing in colour, tannins are astringent, highly diverse polyphenolic biomolecules divided into two classes: hydrolysable tannins (such as gallotannins and ellagitannins) and condensed polyflavonoid tannins. Hydrolysable tannins can only be found in dicotyledons. Among condensed tannins, the most common are procyanidins in the form of catechin and epicatechin, then prodelphinidin tannin in the form of gallocatechin and epigallocatechin, and propelargonidin tannin in the form of afzelechin and epiafzelechin. Conifers are considered as the most abundant tannin source [19,50,52].

The specific chemical structure and the resulting reactivity enable tannins to irreversibly bind to metals and other molecules, including proteins, creating durable complexes [19,50,52]. These properties make them useful for a multitude of applications. For example, they are traditionally used in leather production and applied as additives for beer, wine and fruit juices as antioxidants and flavouring agents [50,51,53,54,55,56]. They can be used for purification of wastewater, production of insulating and fireproof foams, hydroponic horticulture foams, thermoset plastic, resins and flexible plastic films [50,57,58,59]. They can serve as adhesives and surface finishes for wood and wood-based products, cement superplasticisers, anticorrosion coatings for metal, high-temperature resistant surface finishes for metals and Teflon, packaging materials, additives for drilling fluids, to name only a few [50,60,61,62,63].

The already published results of research on potential pharmaceutical and medical applications of tannins point out to their positive effect on the functionality of intestinal as well as to anticancer, anti-inflammatory, anti-allergic or antiviral activity [43,50,51,56,64,65,66,67,68,69]. The specific properties of tannins that enable their irreversible binding to proteins make them also a useful weapon against microorganisms. Several studies confirmed their antibacterial activity; there is also a tannin-based pharmaceutical to cure intestinal infections [50,69,70,71,72,73]. Similarly, the effective activity of tannins against diverse pathogenic fungi species, i.e., dermatophytes, moulds and yeasts, have been reported [74,75,76,77]. Hence the idea to try tannins as antifungal wood preservatives. Since most wood-decaying fungi use extracellular enzymes to degrade wood components, the presence of tannins will result in their inactive complexes with fungal enzymes, thus protect wood against biodegradation [78,79].

#### 2.2.1. Tannins in Wood Protection

Antifungal properties of eight different tannin fractions extracted from Norway spruce bark and cones, and Scots pine cones against eight different brown-rot fungi, three white-rot fungi and four soft-rot fungi species on malt agar medium on Petri dishes were studied by Anttila et al. [76]. Cone tannins were more effective in inhibition of fungal growth than bark tannins. However tannin extracts showed better inhibitory effect against brown-rot than white- or soft-rot species, they were considered as potential substances for wood protection. Similar experiments were performed by Özgenç et al. [80] using maritime (*Pinus pinaster* L.), iron (*Casuarina equisetifolia* L.), mimosa (*Acacia mollissima* L.), Calabrian pine (*Pinus brutia* Ten.), and fir (*Abies nordmanniana*) tree bark extracts against *T. versicolor* and *C. puteana* fungi. Maritime pine and fir bark extracts showed better resistance against *T. versicolor*, while iron and mimosa bark extracts were more effective against *C. puteana*. The conclusion from the study was that the most important factor in antifungal activity is a concentration of an extract. Unfortunately, no particular compounds of the extracts were indicated in this study as the most effective inhibitors of fungal growth.

Several studies were performed to evaluate the resistance of different wood species treated with tannins against moulds and wood-decaying fungi.

Abundant with tannins, water extracts from leaves of Sicilian sumac and valonia oak, and bark of Turkish pine were used by Sen et al. [81] for Scots pine and beech wood treatment. Beech samples were then exposed to white-rot fungus *T. versicolor,* and Scots pine specimens were exposed to brown-rot fungus *G. trabeum*. The most resistant were samples treated with valonia oak extracts. However, the antifungal efficacy of the applied treatment significantly decreased after leaching, which points to the poor fixation of tannins in the wood structure.

Tascioglu et al. [82] studied antifungal properties of tannin-rich bark extracts of mimosa (*Acacia mollissima*), quebracho (*Schinopsis lorentzii*) and pine (*Pinus brutia*) applied for impregnation of Scots pine, beech and poplar wood. The results of mycological tests against two white-rot (*T. versicolor* and *Pleurotus ostreatus*) and two brown-rot fungi (*Fomitopsis palustris* and *G. trabeum*) revealed high antifungal effectiveness of mimosa and quebracho extracts, especially when applied into Scots pine wood. Pine bark extracts (even at a concentration of 12%) were ineffective. The results suggested that mimosa and quebracho extracts can be utilised as environmentally friendly preservatives for wood utilised indoors. Increased activity of mimosa tannin against *T. palustris* and *C. versicolor* was reported by Yamaguchi and Okuda [83] after its chemical modification and removal of low molecular weight compounds by dialysis. Tannin extracts from *Acacia mearnsii* were reported by Da Silveira et al. [84] as an effective wood preservative against white-rot fungus *P. sanguineus.* In turn, Mansour and Salem [85] showed a complete suppression of *T. harzianum* (mould) growth by *Maclura pomifera*, *Callistemon viminalis* and *Dalbergia sissoo* bark extracts.

Valonia, chestnut, tara, and sulphated oak tannins were used by Tomak and Gonultas [86] for impregnation of Scots pine wood. Their antifungal effectiveness against brown-rot *C. puteana* and *P. placenta*, and white-rot fungi *T. versicolor* and *P. ostreatus* was evaluated. The results showed that tannins efficiently suppressed the attack by brown-fungi while were not effective against white-rot. The best antifungal activity was observed for valonia and chestnut tannins, presumably due to the higher ellagitannins content. However, leaching significantly decreased the effectiveness of the applied tannin treatment. Ellagitannins were also indicated by Hart and Hillis [79] as compounds responsible for the resistance of white oak heartwood resistance to *Poria monticola*.

#### 2.2.2. Tannins in Combination with Other Substances

Some attempts have also been made to apply tannins in combination with other compounds with proven antifungal activity, such as boron or copper ions, to increase their performance and enhance their fixation in the wood structure.

Yamaguchi and Okuda [83] used mimosa tannin-copper-ammonia complexes for impregnation of *Cryptomeria Japonica* D. Don wood. The applied treatment resulted in increased resistance to leaching and fungal decay. Improved antifungal efficacy of condensed tannin-containing bark extracts from loblolly pine (*Pinus taeda*) complexed with copper(II) ions applied on birch samples against *C. versicolor* in comparison with bark extracts themselves was confirmed by Laks [78,87]. A similar effect was obtained by Ramirez et al. [88] for *Cocos nucifera* tannin–copper complex solutions applied on alder samples, and for Bernardis and Popoff [89] who reported high resistance of *Pinus elliottii* wood samples treated with “quebracho colorado” tannin extract complexed with CCA salt solution against white-rot *P. sanguineus* and brown-rot fungus *Gloeophyllum sepiarium*.

Research by Thevenon et al. [90] showed enhanced effectiveness of preservative systems based on condensed mimosa tannins, hexamine and boric acid against very aggressive tropical white-rot fungus *P. sanguineus* in comparison with tannin extracts applied alone. The results revealed a decreased leachability of boron while it is complexed in the network of tannins and hexamine. Further study on similar complexed formulations showed their high effectiveness against *C. versicolor* and *C. puteana* while applied on beech, beech plywood and Scots pine wood, respectively [91,92]. They also indicated that increased resistance of boron to leaching results from its covalent fixation in the tannin-hexamine network [91].

In turn, Salem et al. [93] reported high anti-mould effectiveness of a composition of tannin-reach inner and outer bark extracts from sugar maple (*Acer saccharum*) with citric acid when applied on *Leucaena leucocephala* wood. P-hydroxy benzoic acid, gallic acid and salicylic acid were indicated as the main components of biological activity.

The multi-component tannin-based wood preservative systems described above seem to be a promising alternative to artificial fungicides for outdoor applications.

### 2.3. Wood Extractives

Several wood species have a high natural resistance to decay due to the presence of diverse extractable chemical compounds collectively referred to as extractives. Extractives are diverse non-structural wood components produced by trees as defensive agents against environmental stresses and are mainly located in the heartwood. Generally, they can be classified into two different groups: aliphatic and alicyclic compounds (i.e., terpenoids and terpenes) and phenolic compounds (i.e., flavonoids and tannins). Their antifungal effectiveness, depending on the type of the active molecule, can be based on different mechanisms, including direct interaction with fungal enzymes, disruption of cell walls and cell membranes structure leading to leakage of the cell content or disturbance in ion homeostasis, or antioxidant activity [11,94,95].

Naturally durable wood is a valuable material in the market and an environmentally friendly alternative to wood treated with traditional chemicals. Potentially, industrial waste from the processing of durable wood species could serve as a source of natural, commercially viable biocides that can be used for the treatment of less durable wood. Hence extensive research on wood extractives has been carried out worldwide [96,97,98].

Teak (*Tectona grandis* L.f) is one of the known highly durable wood species. However, its resistance to fungal decay varies significantly between trees from different geographical zones, plantations or of different ages. Some results of the studies on the antifungal properties of teak hardwood suggest that they may result from the synergistic effect of various extractive compounds, e.g. anthraquinines and tectoquinones [99,100,101], while other data indicate the role of a single specific compound rather than the total quantity of extractives in determining wood decay resistance [102,103]. Haupt et al. [102], who studied decay resistance of teak wood from Panama, identified tectoquinone as a bioactive compound inhibiting the growth of *C. puteana*. Research by Thulasidas and Bhat [103] reported high resistance of teak heartwood from Kerala (India) against brown-rot (*Polypomus palustris* and *G. trabeum*) and white-rot (*P. sanguineus*, *T. hirsuta* and *T. versicolor*), specifying naphthoquinone as the most important active compound. Anda et al. [100] showed high natural resistance of teak wood from Mexico to white- (*P. chrysosporium*) and brown-rot (*G. trabeum*) fungi, while its resistance against the white-rot fungus *T. versicolor* was only moderate. They identified tectoquinone, deoxylapachol, isolapachol and dehydrotectol as the supposed components responsible for wood durability. Mycological tests carried out by Kokutse et al. [99] showed that teak wood from Togo was highly resistant to *P. sanguineus* and *G. trabeum*, while <20% wood mass loss was reported after wood exposure to *Antrodia* sp. and *C. versicolor*. Brocco et al. [98] showed the effectiveness of ethanol extracts from waste materials obtained in mechanical processing of teak heartwood from Brazil in the protection of treated teak and pine sapwood against white- and brown-rot fungi. No antifungal activity against soft-rot was observed.

Kirker et al. [97] studied the natural resistance of several wood species obtained from different lumber producers in North America to selected brown- and white-rot fungi. Their results showed high durability of coniferous species such as eastern red cedar, western juniper, western red cedar and Alaskan yellow cedar, as well as deciduous black locust, honey mesquite and catalpa. Southern pine and paulownia wood occurred the less resistant to decay. The extractives of paulownia wood had no or marginal inhibitory effect on *T. palustris* and *G. trabeum* and honey mesquite extractives were not effective against *I. lacteus*. Füchtner et al. [104] showed that the resistance of non-durable Norway spruce heartwood to brown-rot fungus *R. placenta* results from the presence of fungitoxic hydrophobic resin, while in the case of moderately durable Kurile larch heartwood, the resistance is due to large amounts of different antioxidant flavonoids.

Sablík et al. [96] reported efficacy of black locust (*Robinia pseudoacacia* L.) heartwood extracts to increase decay resistance of non-durable European beech (*Fagus sylvatica* L.) wood from class 5 (not durable, mass loss about 44%) to class 3 (moderately durable, mass loss about 13%). Whereas extractives from *Dicorynia guianensis* Amsh heartwood from French Guyana were shown by Anouhe et al. [105] to have antifungal activity against *P. sanguineus* and *T. versicolor* mainly due to the presence of alkaloid compounds.

Extracts from the xylem of *Cinnamomum camphora* (Ness et Eberm.), which is a Chinese hardwood species, were tested by Li et al. [106] against two wood-rot fungi: *G. trabeum* and *Coriolus (Trametes) versicolor*. The best results were obtained for chloroform and methanol extracts, where effective dose for 50% growth inhibition was 7.8 mg/mL of chloroform extract against *C. versicolor* and 0.3 mg/mL of methanol extract against *G. trabeum*. The most abundant components of both extracts with proven antifungal activity were camphor and α-terpineol. *C. camphora* then can be considered as a source of natural antifungal preservatives for wood protection.

The anti-mould activity of heartwood extracts has also been studied. Maoz et al. [107] showed that however extracts from the wood of Alaska cedar, Western juniper, incense cedar and Port Orford cedar can reduce the growth of mould (*Paecilomyces*, *Trichoderma*, *Penicillium*, *Aspergillus*, *Graphium* and *Sporothrix* species) on Douglas-fir sapwood, they are not able to completely protect wood against fungi. Therefore, only multi-component extracts can be considered as potential alternatives for traditional wood protection systems. Effectiveness of wood extracts against mould was also studied by Mansour and Salem [85]. They reported a complete suppression of *T. harzianum* growth by *Cupressus sempervirens* L. and *Morus alba* L. wood extracts at the concentration of 1000 mg/mL, showing the potential of local wood extracts (Egypt) as an anti-mould biocide. The results of another study by Salem et al. [108] indicated good resistance of Scots pine (*P. sylvestris* L.), Pitch pine (*P. rigida* Mill.), and European beech (*Fagus sylvatica* L.) wood treated with *Pinus rigida* heartwood extracts against several mould fungi (*Alternaria alternata*, *Fusarium subglutinans*, *Ch. globosum*, *A. niger*, and *T. viride*). However, the applied *P. rigida* heartwood methanol extract did not completely reduce fungal growth. Its main constituents were identified as α-terpineol, borneol, terpin hydrate, D-fenchyl alcohol, and limonene glycol.

The most common problems with wood extractives applied for the antifungal treatment of low-durable wood are their diversity and inconsistency in their biological activity, as well as problems with leachability from wood. To overcome the latter, their fixation onto the wood surface using an enzyme-mediated reaction was proposed as a green alternative to traditionally used chemicals [109].

### 2.4. Other Plant Extracts

Besides essential oils, tannins and wood extracts, there are several other substances of plant origin, derived from different parts of a plant using various methods, with proven antifungal properties that could potentially be applied to enhance wood resistance against fungal attack.

Tea and coffee are one of the most economically valuable crops worldwide. Their health benefits have been known to man for centuries. Among other biologically active secondary metabolites playing an important role in plant protection against pathogens, they contain caffeine —an alkaloid that exhibits i.e., antioxidant, antimicrobial, immunologic, anti-cancer, but also antifungal properties [110,111,112]. Tea and coffee extracts were tested against wood-inhabiting fungi to evaluate their potential effectiveness in wood protection. In general, green tea extracts exhibited a higher inhibitory effect on selected white-, brown- and soft-rot fungi than coffee, orthodox black tea and commercial black tea extracts. However, filtration removed most of the biologically active compounds from the extracts. White-rot fungi were the most sensitive among all the tested species. The main constituent of tea and coffee extracts, caffeine, proved high inhibitory effect on most of the tested fungi [113]. Similar results were obtained using tea extracts and caffeine against tea-plant fungal pathogens, confirming fungicidal effectiveness of the latter [114]. It was shown that the mechanism of caffeine fungistatic activity involves its damaging effect on the fungal cell wall and cell membrane [112]. Another study focused on the potential antifungal effectiveness of coffee silverskin, which is a waste product in the industrial process of coffee roasting. It turned out that coffee silverskin hot water extracts contain chlorogenic acid and caffeine derivatives able to inhibit the growth of *Rhodonia placenta*, *G. trabeum* and *T. versicolor*. Moreover, their ecotoxicity was significantly lower in comparison with commercial copper-based wood preservative, making them a potential feedstock for obtaining chemicals useful in wood preservation [115]. Pure caffeine solutions applied on Scots pine samples effectively reduced wood susceptibility to mould (*A. niger*, *A. terreus*, *Ch. globosum*, *Cladosporium herbarum*, *Paecilomyces variotii*, *Penicillium cyclopium*, *P. funiculosum*, *T. viride*), brown-rot fungi *C. puteana* and *P. placenta*, and white-rot fungus *T. versicolor*. However promising in wood protection against fungi, caffeine turned out to be easily leachable from wood, which is its main disadvantage precluding it from application for wood used outdoors [116]. Therefore, several attempts have been made to stabilise caffeine inside the wood structure using organosilicon compounds [117] or a mixture of silanes and propolis [118].

Low concentrations of extracts from poisonous *Nerium Oleander* L. were shown by Goktas et al. [119] as effective in the protection of Turkish oriental beech and Scots pine wood samples against brown- and white-rot fungi *P. placenta* and *T. versicolor*, respectively. Similar properties were also reported for extracts of *Gynadriris sisyrinchium* (L.) Parl, another poisonous plant [120]. Also, lichen (*Usnea filipendula*) and mistletoe (*Viscum album*) leave extracts applied on Scots pine sapwood reduced wood susceptibility to the fungal attack of *C. puteana* [121].

Pyrolysis distillate components were studied by Barbero-López [122] as a potential alternative resource for wood preservative agents. Hemp, birch and spruce distillates at a concentration of 1% inhibited the growth of *C. puteana, R. placenta* and *G. trabeum*. Propionic acid was identified as the most effective antifungal compound. In turn, Sunarta et al. [123] reported high antifungal effectiveness of a bio-oil obtained from pyrolysis of palm fruit shell against blue stain fungus *Ceratocystis* spp.

Moderate anti-mould properties of 3% water extracts of *Acacia saligna* (Labill.) H. L. Wendl. flowers were reported by Al-Huqail et al. [124] when applied on *Melia azedarach* wood samples, showing its potential for wood preservation. Among the main active compounds with proved antifungal properties were benzoic acid, caffeine, naringenin and quercetin. Extracts of *Withania somnifera* fruit significantly limited mycelial growth of *A. alternata*, *Bipolaris oryzae*, *Colletotrichum capsici*, *C. lindemuthianum*, *Curvularia lunata*, *Fusarium culmorum*, *F. oxysporum*, *F. moniliforme*, *Macrophomina phaseolina*, *Rhizoctonia solani*, and *Pyricularia oryzae*, showing their potential in antifungal protection of plants and wood [125,126,127]. Antifungal activity of theses extracts was attributed to the single or the synergistic effect of several compounds, including alkaloids, flavonoids, glycosides, saponins or tannins. Bi et al. [128] in turn studied decay resistance of poplar wood treated with ethanol extracts of konjac (*Amorphophallus konjac* K. Koch) powder. The extracts were more effective against brown-rot *G. trabeum* than white-rot *T. versicolor*. Salicylic acid, vanillin, 2,4,6-trichlorophenol and cinnamaldehyde were identified as the most active compounds.

Some leaves extracts were also reported to possess antifungal activity against wood-inhabiting fungi. They can be an economically viable potential source of bio-friendly wood preservatives due to the fact that they can be easily obtained directly from trees or as a by-product during forest harvesting. Maoz et al. [107] showed the effectiveness of Alaska cedar, Douglas-fir, western red-cedar and Pacific silver-fir leaves extracts in the protection of treated Douglas-fir sapwood against mould attack of *Trichoderma* and *Graphium* species. Collective ethanol extracts from root, stem and leaves of *Lantana camara*, reach in alkaloids, terpenoids and phenolics, completely inhibited the growth of white-rot *T. versicolor* and brown-rot *Oligopous placentus* [129]. Methanol extracts of *Magnolia grandiflora* L., as shown by Mansour and Salem [85], affected the growth of a common wood mould pathogen *T.a harzianum*, while *Robinia pseudoacacia* leaves extracts effectively inhibited the growth of wood-decaying fungus *T. versicolor* [130].

## 3. Antifungal Substances of Animal Origin

Several compounds of animal origin have been already used in wood protection. Waxes (beeswax) were applied mainly to increase water resistance and protect wood against photochemical degradation. Biopolymers, such as gelatin, zein or other proteins, were used as components of wood protective coatings and adhesives, enhancing moisture resistance and dimensional stability, and preventing leachability of biocides from wood [16,131,132,133,134,135]. However, some of them occurred also to possess direct antifungal properties and potentially could be used alternatively to traditional fungicides.

### 3.1. Propolis

Propolis, also known as bee glue, is a natural resinous substance that is synthesised by honeybees from products harvested from tree buds and other plant exudations mixed with their saliva, bee enzymes, beeswax and pollen. A waxy nature and good mechanical properties make propolis a perfect insulation material enabling to keep a constant temperature and moisture content inside the hive throughout the whole year. It is used to reinforce the structural stability and smooth out internal walls of the nest, as well as to seal small holes and cracks in the hive or honeycombs. Propolis provides antibacterial and antifungal protection to the nest, and serves to cover carcasses of intruders who find its way into the hive and die inside, and are too big for bees to be carried out, avoiding their putrefaction inside. Overall, propolis is used for hive defence, hence its name derived from Greek and originated from the words “pro”, which stands for “at the entrance to” or “in defence”, and “polis”, which means “city” [136,137,138,139,140,141].

At temperatures over 20 °C, propolis is a soft, pliable and sticky substance. When cooled, it becomes hard and brittle. Its colour is usually dark brown, but it also can occur in black, red, yellow, green or white hues, depending on the botanical source [137,142,143,144]. Generally, it is a complex mixture that contains 50% of resins and balsams, 30% of wax, 10% of essential and aromatic oils, 5% of pollen and 5% of impurities [138,140,144]. The chemical composition of propolis differs considerably between particular hives, bee species, regions and seasons mainly due to the variability of plant species growing around and being a source of exudations collected by bees [137,138,140,141]. By now, more than three hundreds chemical constituents have been identified, mainly including polyphenols (flavonoids, phenolic acids and their esters), terpenoids, steroids, amino acids, aromatic compounds, volatile oils and bee wax [140,141,144].

Since ancient times, propolis has been applied for a variety of purposes. Several civilisations used it in traditional medicine, for example, to cure colds or heal wounds. Ancient Greeks applied it as an antiseptic for cutaneous and buccal infections, while Egyptians used it for embalming dead bodies [137,138]. Due to its antimicrobial, antioxidant, antiviral, anti-inflammatory, anti-tumour and immunomodulatory activities provided mainly by phenolic compounds, it is still used in folk and complementary medicine as an almost universal cure [137,140,145,146].

In recent times, composition and properties of propolis have been extensively studied worldwide, confirming its usefulness in multiple therapeutic applications, as well as an ingredient in superfood and biocosmetics. Although standardisation of its chemical composition remains challenging, the presence of numerous molecules with many useful properties is undeniable [137,138,139,140,147,148]. Antibacterial properties were attributed to caffeic acid, diterpenic acids, ferulic acid, *p*-coumaric acid, galangin, lignans, pinocembrin and syringe aldehyde. Antiviral activity was assigned to caffeic acid and its derivatives, kaempferol, *p*-coumaric acid and quercetin. The antifungal activity was shown for (+)-agathadiol, benzoic acid, caffeic acid and its ester, ferulic acid, *p*-coumaric acid, benzyl ester, epi-13-torulosol, galangin, isocupressic acid, pinobanksin, pinocembrin, sakuranetin and pterostilbene [141,148,149,150,151,152,153,154,155].

#### 3.1.1. Propolis in Wood Protection

Although propolis has been used for thousands of years for various purposes, its application for wood treatment is hardly known. The only exceptions are information about top-class violin makers, including Stradivarius and masters from Cremona in Italy. They applied propolis-based varnish invented by themselves to polish their instruments for enhancing their acoustic properties or used it as a mixture with other ingredients as a colouring or finishing coat [149,156]. Nowadays, propolis has been tried for wood finishing individually or in a mixture with silanes. The results show that even though its effect on wood properties was mediocre, it could be a welcome addition to wood finishes based on natural ingredients [149,157,158]. Due to the proven antifungal properties, however, propolis has also been conceived as a potential natural and an environmentally-friendly wood preservative against moulds and wood-decaying fungi [150,159,160,161,162].

#### 3.1.2. Propolis Activity against Mould

Antifungal activity of propolis from Argentina against several phytopathogenic moulds, including those occurring in wood, such as *A. niger*, *Trichoderma* spp., *Penicillium notatum* or *Fusarium* sp., was evaluated by Quiroga et al. [150]. They examined a partially purified ethanol extract of propolis as well as two of its flavonoid components isolated by HPLC—pinocembrin and galangin. Their results clearly show that both propolis and its isolated components were effective against the tested fungi and were characterised by low cytotoxicity. This means that propolis is safe for the environment and can be applied as an antifungal agent to protect other natural products, including wood, against mould. Anti-mould effectiveness of propolis from the US and China against *P. notatum*, with the main components such as pinocembrin, pinobanksin-3-*O*-acetate, galangin, chrysin, pinobanksin, and pinobanksin-methyl ether, was also confirmed by Xu et al. [163].

#### 3.1.3. Propolis Activity against Wood-Decaying Fungi

Extracts of propolis from all around the world or their particular ingredients were used for impregnation of wood of different species to examine their potential in wood protection against wood-decaying fungi.

Woźniak et al. showed that ethanol extracts of Polish propolis of concentration exceeding 12% limited effectively decay of Scots pine wood by *C. puteana* [161]. The higher the propolis content in a solution was, the better antifungal effect was achieved, reaching a wood mass loss of 5.9%, 3.3%, 2.3% and 2.7% for propolis concentration of 7.5%, 12%, 18.9% and 30%, respectively. Moreover, high concentrations of three flavonoids known for their antifungal activity were identified in Polish propolis extracts: pinocembrin, galangin and chrysin (about 47, 29 and 23 mg/g, respectively).

Scots pine and paulownia wood treated with 7% methanol extract of Turkish propolis were more resistant to *Neolentinus lepideus* (brown-rot) and *T. versicolor* (white-rot) compared with untreated samples. For Scots pine, mass loss was 29.7% and 2.5% for untreated and treated wood exposed to *N. lepideus*, and 28.4% and 4.2% for untreated and treated wood exposed to *T. versicolor*, respectively. In the case of low-durable paulownia wood, however, the results were not that good, with mass loss of 39.2% for untreated and 12.3% for treated wood exposed to *T. versicolor*, and 47.2% for untreated and 11.6% for treated samples exposed to *N. lepideus* [159].

Budija et al. [158] demonstrated that an ethanol extract of propolis 29% from Eastern Slovenia effectively protected Norway spruce wood against brown-rot fungi *Antrodia vaillantii* and *G. trabeum*, and a white-rot fungus *T. versicolor*, resulting in wood mass loss of 5.3%, 7.2% and 4.6%, respectively. Also, poplar wood treated with a propolis solution of 40 mg/mL was more resistant to *T. versicolor* than untreated wood (mass loss of about 11% vs 20%, respectively, after eight-weeks exposure) [162]. However, in this case, a gradual decrease of antifungal effects of propolis was observed over time during exposure to fungi. This may result from biodegradability of the particular propolis ingredients or low retention of the propolis solution into wood, which are widespread drawbacks of natural biocides.

Ethanol extract of propolis from Argentina, as well as its isolated compounds pinocembrin and galangin, proved to effectively inhibit fungal hyphal radial growth of white-rot fungi *P. sanguineus* and *S. commune* and were slightly less effective against *Ganoderma applanatum* and *Lenzites elegans*, showing their potential in wood protection against decay [150].

Jones et al. [40] treated samples of different wood species with methanol or aqueous soda solutions of propolis commercially available in health shops in the UK. They exposed them to wood-decay fungi *C. puteana* and *P. placenta*. Their results proved excellent resistance of the treated wood against *C. puteana* and slightly lower protection against *P. placenta.* However, the protective effect was more pronounced for Scots pine, ash and larch than for Western red cedar or Sitka spruce wood. Unfortunately, the experiments also showed high sensitivity of the propolis treatment to leaching, therefore it cannot be applied for outdoor purposes without additional fixation in wood.

#### 3.1.4. Propolis in Combination with Polymers

The observed shortcomings of propolis extracts applied as wood preservatives, such as leachability from wood and a gradual decrease in antifungal activity over time [40,162], prompted researchers to search for stabilisers that would enhance the effectiveness of propolis. In wood preservation, application of some polymers, such as proteins or organosilicon compounds, proved effective in retaining fungicides in wood [14]. A similar approach was successfully applied for propolis. Woźniak et al. showed that a mixture of propolis extract with organosilicon compounds methyltrimethoxysilane and vinyltrimethoxysilane was more effective in protecting Scots pine wood against *C. puteana* than the propolis extract used alone. Instead, Ratajczak et al. proved that Scots pine wood treated with a formulation based on propolis, caffeine, methyltrimethoxysilane and octyltriethoxysilane was resistant to *C. puteana* even after the accelerated ageing procedure that involved leaching [118].

Results presented herein show the potential of propolis in wood protection against fungi. However, due to the challenges such as high variability of the propolis composition and the problems with its sustainability when applied to wood, its early introduction to the market as a ready to use product seems impossible without the improvement of its performance. Further research is then necessary,

### 3.2. Chitin and Chitosan

Chitin is a natural, white, hard, inelastic mucopolysaccharide consisting of 2-acetamido-2-deoxy-β-d-glucoses linked by β(1→4) bonds. Abundant in nature, it is the main component of exoskeletons of arthropods, including marine crustaceans such as shrimp and crabs, cell walls of fungi, spines of diatoms or the scales of fish. It is structurally comparable to cellulose, with similar low solubility and low chemical reactivity [164,165,166]. Chitosan is the *N*-deacetylated derivative of chitin. Its production is economically feasible since its main source is crustacean shells obtained as a food industry waste. Renewable, biodegradable, biocompatible and non-toxic, chitin and chitosan have recently gained particular attention as potential natural polysaccharide resource useful for manufacturing of many value-added products. Due to their anti-cancer, antioxidant, anticoagulant and antimicrobial properties, they are applied for production of drug carriers, artificial skin and bones, wound dressings, contact lenses, solid-state batteries. They are also used as chelating agents for wastewaters purification, and as additives for food, cosmetics and paper making [164,165,166,167,168,169].

Chitosan proved to have also fungicidal and fungistatic activity [164,170,171]. However, its high diversity in terms of chemical structure makes it difficult to determine its antimicrobial properties precisely. The most important factors that play a role in biocidal action are the molecular weight, the degree of deacetylation and polymerisation of chitosan as well as the type of microorganism [168,170,172]. Chitosan proved to interact with the fungal cell wall and alter its structure, and two types of mechanisms behind the antimicrobial activity of chitosan have been found already [14,173,174]. One of them involves permeabilisation of the plasma membranes of bacteria or fungi due to electrostatic interactions between amino groups in chitosan chain and molecules in the cell surface, leading to leakage of intracellular material and death of the cell [171,172,174,175,176,177]. The second relates to changes in gene expression by interactions between chitosan and nucleic acids [171,178,179,180].

Antifungal properties of chitin and chitosan are successfully utilised not only in the food and cosmetics industry but also have a high potential in agriculture since they are useful in the protection of plants against fungal pathogens and an extension of the commercial life of fruit [166,181,182,183,184]. Hence the idea to apply this substance for the preservation of another natural material that is wood, to protect it against mould and decay.

#### Chitosan in Wood Protection

Many attempts have been made to evaluate the effectiveness of chitosan in wood protection against fungi. Experiments performed in agar plates showed that the fungal growth rate decreased with an increase in chitosan concentration and molecular weight, whereby no apparent difference was seen between mould, white- and brown-rot fungi [185,186,187,188,189]. Generally, 1% chitosan solution totally inhibited fungal growth [188,190].

Application of chitosan into wooden blocks revealed its potential as an antifungal agent. Kobayashi et al. showed that Sugi wood treated with chitosan (uptake of 11.6 kgxm^−3^) was more resistant to brown-rot *T. palustris* and white-rot *T. versicolor* fungi (mass loss of 15.9% and 4.9%, respectively) than untreated wood (mass loss of 34.8% and 19.7%) [191]. Also *Fagus crenata*, *Pinus densiflora* and *Cryptomeria japonica* wood treated with chitosan were more resistant to soil microorganisms and decay fungi (*C. versicolor*, *T. palustris*, *S. lacrymans*) in comparison with untreated wood [192].

Schmidt et al. reported an increased resistance of Scots pine wood treated with chitosan solution with the uptake of 5.6–6.8 kgxm^−3^ to brown-rot *C. puteana* and *G. trabeum*, with average mass loss of 1.6–3.2% and 3.7–6.0% in comparison with 18.2% and 35.6% for untreated control, respectively [193]. Eikenes et al. obtained similar results for Scots pine mini blocks treated with 4.8% (*w*/*v*) solution of high molecular weight chitosan, exposed to *C. puteana* and *P. placenta*. The reported mass loss was 1.6% and 0.1% for treated wood compared to 60% and 35% for untreated samples, respectively [188]. However, some elution of chitosan was observed after accelerated leaching of the treated samples in water. It was the more pronounced, the lower molecular weight of chitosan was. Nevertheless, 5% chitosan solution proved effective against decay fungi despite leaching [188]. Alfredsen et al. and Gorgij et al. confirmed higher effectiveness of high molecular weight chitosan against mould and blue stain compared to that of low molecular weight [190,194].

In turn, Larnøy et al. reported antifungal effectiveness of 5% solution of low molecular chitosan used for Scots pine and beech treatment [195]. Average mass loss of treated Scots pine exposed to *C. puteana* and *P. placenta* was 4.9% and 1.6% compared to 37.7% and 42.7% for untreated samples, respectively. The mass loss for treated beech wood exposed to *T. versicolor* was 2.8% compared to 30.2% for untreated wood after eight weeks of accelerated decay test.

The results of the application of chitosan on historical wood samples by El-Gamal et al. demonstrated the effectiveness of the treatment against mould and confirmed that it could be recommended for the protection of archaeological wooden objects [196].

Chitosan can form a membrane inside the wood structure that not only acts as a barrier against moisture and air but can also retain other particles and prevent their leaching from wood [195,197]. Therefore, an attempt was made to apply it in combination with metals with antifungal properties or fungicides. It was successfully used with copper, zinc, silver, chromated copper arsenate-based preservatives or tebuconazole, proving effective wood protection against mould and decay [191,198,199,200].

## 4. Conclusions

As can be seen, natural compounds have enormous potential in wood protection since they exhibit a broad spectrum of antimicrobial activities. They are renewable, readily available or cost-effectively obtainable from waste materials, non-toxic or of much lower eco-toxicity than traditional chemical biocides, and environmentally-friendly. However, they also have some limitations, including high heterogeneity depending on the source from which they are derived (i.e., propolis, essential oils, wood extractives), lack of appropriate retention inside the impregnated wood tissue, easy leachability, selective or uneven activity against particular types of fungi, high susceptibility to biodegradation. Some of these problematic issues seem to be surmountable by combining organic biocides with:

- different biological compounds able to degrade pit membranes thus increasing their penetrability into wood tissue;
- various natural polymers and cross-linkers to fix natural compounds inside the wood structure and prevent their leaching;
- other substances such as antioxidants, biological control agents or chelators to enhance their antimicrobial activity and durability.

Market launch of natural biocides is additionally hindered by some discrepancies between laboratory tests and field performance reported, as well as legislation-related problems due to the necessity to meet the requirements of various directives (related to construction materials and application of biocides) and lack of standards defining the quality, composition, performance and application of particular natural-based protective formulations. Therefore, further research in the field is necessary.

Since responding to all the challenges that face the development of natural preservatives oriented specifically towards the protection of wood and wood-based products may turn out to be too costly to be profitable, combining forces with other branches of industry interested in the exploitation of particular natural active compounds (i.e., crop protection, pest control, food and pharmaceutical applications) might prove to be a good solution.

Nowadays, when the extending lifespan of wood products is of great interest and importance, developing new generation natural preservatives with minimal impact at the end of stage life of the treated wood is an imperative from the perspectives of human health and environmental protection. Although the presented review does not exhaust the subject, since there are hundreds of scientific data concerning the antifungal activity of natural substances, it gives a comprehensive view on the current state of research in the field and shows the prospects of the development of sustainable alternative wood protection based on natural compounds.

## Figures and Tables

**Table 1:** The main types of fungi that can colonise and degrade wood [1,2,3,4,5].

Type of Fungi	Degraded Wood Type and Components	Effect on Wood
**Wood-decaying fungi**		
brown-rot (Basidiomycota)	mainly softwoods; degradation of hemicelluloses and cellulose, demethylation of lignin	wood shrinkage and cracking into cubical pieces, brown colouration due to the presence of lignin remained, reduction of wood mechanical properties
white-rot (Basidiomycota)	mainly hardwoods but also softwoods; degradation of lignin and hemicelluloses, but also cellulose	fibre-like appearance and white colouration of wood due to the presence of lighter-coloured cellulose remains, wood becomes soft and spongy or stringy, its strength properties decrease along with the decay progress
soft-rot (Ascomycota, fungi imperfecti)	hemicelluloses and cellulose, less extensively lignin	formation of cavities inside the cell wall, discolouration and cracking pattern similar to brown-rot, deterioration of wood strength properties
**Mould**		
mould (Zygomycota or Ascomycetes)	easily available sugars, not structural polymers	superficial discolouration of wood, minor degradation of the wood surface
**Blue stain**		
blue stain (Ascomycota and Deuteromycota)	protein content of the parenchyma cells, easily available sugars, not structural polymers	dark discolouration of sapwood by dark-coloured hyphae, degradation of pit membranes leading to increased water permeability
